# ANTIBIOTIC PROPHYLAXIS FOR ABDOMINAL SURGERY: WHEN TO RECOMMEND? BRAZILIAN COLLEGE OF DIGESTIVE SURGERY POSITION PAPER

**DOI:** 10.1590/0102-672020230040e1758

**Published:** 2023-09-15

**Authors:** Alexandre Coutinho Teixeira de FREITAS, Álvaro Antonio Bandeira FERRAZ, Leandro Cardoso BARCHI, Ilka de Fátima Santana Ferreira BOIN

**Affiliations:** 1 Surgical Clinics Unit, Universidade Federal do Paraná – Curitiba (PR), Brazil; 2 Universidade Federal de Pernambuco, Department of Surgery – Recife (PE), Brazil; 3 São Leopoldo Mandic, Faculty of Medicine – Campinas (SP), Brazil; 4 Universidade Estadual de Campinas, Liver Transplant Unit, Department of Surgery – Campinas (SP), Brazil

**Keywords:** Antibiotic prophylaxis, Surgical wound infections, Postoperative complications, Digestive system surgical procedures, Antibioticoprofilaxia, Infecção da ferida cirúrgica, Complicações pós-operatórias, Procedimentos cirúrgicos do sistema digestório

## Abstract

**BACKGROUND:**

Surgical antibiotic prophylaxis is an essential component of perioperative care. The use of prophylactic regimens of antibiotics is a well-established practice that is encouraged to be implemented in preoperative/perioperative protocols in order to prevent surgical site infections.

**AIMS:**

The aim of this study was to emphasize the crucial aspects of antibiotic prophylaxis in abdominal surgery.

**RESULTS:**

Antibiotic prophylaxis is defined as the administration of antibiotics before contamination occurs, given with the intention of preventing infection by achieving tissue levels of antibiotics above the minimum inhibitory concentration at the time of surgical incision. It is indicated for clean operations with prosthetic materials or in cases where severe consequences may arise in the event of an infection. It is also suitable for all clean-contaminated and contaminated operations. The spectrum of action is determined by the pathogens present at the surgical site. Ideally, a single intravenous bolus dose should be administered within 60 min before the surgical incision. An additional dose should be given in case of hemorrhage or prolonged surgery, according to the half-life of the drug. Factors such as the patient’s weight, history of allergies, and the likelihood of colonization by resistant bacteria should be considered. Compliance with institutional protocols enhances the effectiveness of antibiotic use.

**CONCLUSION:**

Surgical antibiotic prophylaxis is associated with reduced rates of surgical site infection, hospital stay, and morbimortality.

## RESUMO

**RACIONAL:**

A antibioticoprofilaxia é um componente importante dos cuidados perioperatórios.

**OBJETIVOS:**

Abordar os principais aspectos da antibioticoprofilaxia em cirurgia digestiva.

**RESULTADOS:**

Ela é definida como a redução da carga de bactérias no sítio operatório através da obtenção de níveis séricos de antibiótico acima da concentração inibitória mínima no momento da incisão cirúrgica. Está indicada em cirurgias limpas com próteses e nas quais a consequência de uma eventual infecção seja grave, bem como em todas as cirurgias limpas-contaminadas e contaminadas. O espectro de ação do antibiótico deve ser de acordo com a flora esperada no sítio cirúrgico e deve ser administrado 60 minutos antes da incisão, em bolus, por via endovenosa e preferencialmente em dose única. Nos casos de hemorragia importante ou cirurgias mais longas, uma nova dose pode ser administrada. O peso do paciente, a história de alergia a medicamentos e a possibilidade de colonização por bactérias multirresistentes devem ser levados em conta. A aderência a protocolos institucionais aumenta a chance de uso adequado da antibioticoprofilaxia.

**CONCLUSÕES:**

A antibioticoprofilaxia está associada à redução das taxas de infecção do sítio cirúrgico, tempo de internação e morbidade.

## Summary of the main recommendations

- Surgical antibiotic prophylaxis reduces the rates of surgical site infections and mortality.
- It is indicated for high-risk clean surgeries, clean-contaminated surgeries, and contaminated surgeries.
- Antibiotic spectrum should be selected according to the local flora of the surgical site. Cefazolin is the most used antibiotic for surgical prophylaxis.
- The dose should be administered within 60 min before the surgical incision. However, some antibiotics should be administered 2 h before the surgical incision.
- Only one dose is usually sufficient for most cases. Additional doses must be given if the procedure lasts more than two antibiotic half-lives or in case of extensive hemorrhage, defined as more than 1500 mL in adults and 20 mL/kg in children.
- Prophylactic antibiotic administration up to 24 h is also acceptable.
- The best route of administration is intravenous. In most cases, the dose is given as a bolus. However, there is some evidence suggesting that continuous infusion is superior.
- The patient’s weight and history of antibiotic allergies should be considered when selecting the appropriate dose and antibiotic.
- Adherence to institutional protocols increases the effectiveness of antibiotic use.
- Resistant bacterial colonization, especially in the case of methicillin-resistant Staphylococcus aureus, should be considered.

## INTRODUCTION

Perioperative care is as important as good surgical skills and technique. Therefore, many multimodal perioperative care protocols have been used worldwide^19,30,33^. These protocols encompass strategies such as nutrition, epidural or regional anesthesia, pain control, minimally invasive techniques, and aggressive postoperative rehabilitation. The central idea is to minimize surgical trauma and reduce risks, which results in reduced morbidity, mortality, costs, and hospital stay^6,18,19^. Antibiotic prophylaxis is also included in these protocols.

The benefits are unquestionable and have been clearly described in the literature. A study that included 49,000 patients from 21 meta-analyses, only from RCT, showed that antibiotic prophylaxis in surgery provides a remarkable reduction in surgical site infection (SSI), regardless of the wound contamination and type of procedure^7^.

However, inappropriate antibiotic use is very common. The main causes of inadequacy are excessive duration of antimicrobial prescription and failure in proper indication^3^. Many problems can arise as a result of this, with the most common being higher SSI rates, bacterial resistance, and *Clostridium difficile* infection^34^.

The primary sources of information regarding surgical antibiotic prophylaxis are the World Health Organization Guidelines, the Guidelines of the Centers for Disease Control and Prevention, and the recommendations of the Surgical Infection Society published by the American College of Surgeons^1,4,5^.

This position paper focuses on the most important aspects of antibiotic prophylaxis in abdominal surgery.

### What is the definition of surgical antibiotic prophylaxis, and what are its benefits?

The purpose of antibiotic prophylaxis is to prevent SSIs by reducing the microbial load at the operation site. To achieve this, the antibiotic must reach effective serum and tissue concentrations, specifically above the minimum inhibitory concentration of the antibiotic at the time of the initial skin incision^5^.

**Figure 1.**
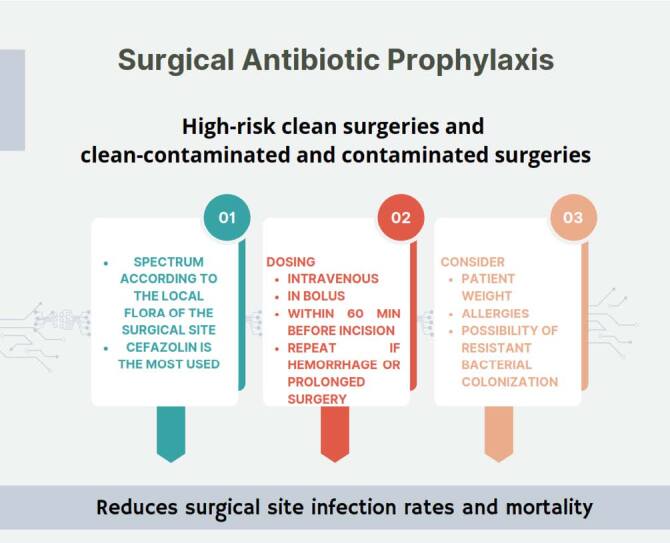
Surgical antibiotic prophylaxis.

The main benefit is to prevent or reduce the risk of SSI. This condition is associated with higher mortality, longer ICU stays, prolonged hospital stays, higher rates of hospital readmission, and increased costs^31^. SSI is defined as the infection of the incision, organ, or space cavity following a surgical procedure. The infection of the incision is considered superficial when it involves the skin and subcutaneous tissue, and it is considered deep when it involves the fascia and muscle. It typically occurs within 30 days of the operation, or within 90 days in cases where prosthetic material was used. There are several risk factors associated with SSI, including wound classification, comorbidities, obesity, age, immunosuppression, and ASA classification^40^. The risk is also influenced by appropriate surgical technique and perioperative care. Wound classification refers to the level of contamination of a surgical wound during the operation^47^. It is considered the primary risk factor for SSI. A clean wound does not involve the respiratory, alimentary, genital, or urinary tracts. It is free from infection and inflammation. A clean/contaminated wound involves the respiratory, alimentary, genital, or urinary tracts under controlled conditions. A contaminated wound can occur due to a significant breach in sterile technique, substantial leakage from the gastrointestinal tract, acute non-purulent inflammation, infarcted or necrotic bowel (non-perforated), and fresh traumatic wounds. A dirty/infected wound exhibits purulence or an existing clinical infection, perforated viscera, devitalized tissue, or it is an open traumatic wound lasting more than 4 h (Figure 1).

The risk of SSI, based on wound classification, is presented in Table 1 ^34,47^.

**Table 1.** Risk of surgical site infection according to the wound classification

Wound classification	Risk of surgical site infection (%)
Clean	1.3–2.9
Clean/contaminated	2.4–7.7
Contaminated	6.4–15.2
Dirty/infected	7.1–40

According to a European report, the risk of SSI is 10.1% for open colectomy, 6.4% for laparoscopic colectomy, and 3.9% for open cholecystectomy^28^.

### When is antibiotic prophylaxis indicated?

It is indicated in two situations. The first situation includes surgeries with serious consequences if infection occurs^47^. The second situation involves surgeries with a high risk of infection^47^. Surgeries with serious consequences in the event of an infection are those where the likelihood of infection is low, but if it does occur, the consequences are associated with high morbidity and mortality. This category includes certain clean surgeries, such as cardiac surgery, neurosurgery, surgeries in immunocompromised patients, and procedures involving the use of prosthetic materials. Clean/contaminated and contaminated surgeries are also considered to have a high risk of infection, requiring antibiotic prophylaxis. It is important to note that in the case of an infected surgery, the concept of antibiotic prophylaxis does not apply. In such cases, the use of antibiotics is considered therapeutic.

### Which antibiotics should be used and what is the appropriate dosing?

Surgical-site pathogens determine the antibiotic spectrum, which means that antibiotic selection is primarily based on efficacy and safety. The choice of antibiotics varies depending on the organ being operated on. In clean surgeries, which do not involve the respiratory, alimentary, genital, or urinary tracts, the antibiotic should cover gram-positive bacteria commonly found on the skin, such as *Staphylococcus aureus* and coagulase-negative *Staphylococcus* ^5^.

In abdominal clean/contaminated and contaminated surgeries, the bacterial spectrum varies and includes gram-positive cocci, gram-negative rods, and anaerobes. In general, more proximal segments of the gastrointestinal tract require coverage for gram-positive bacteria, while more distal segments require coverage for gram-positive, gram-negative, and anaerobic organisms. The most widely used antibiotics are first- and second-generation cephalosporins, such as cefazolin, cefuroxime, cefoxitin, or the combination of cefazolin plus metronidazole. Cefazolin is the drug of choice for most procedures. It has been extensively studied and has demonstrated efficacy, a favorable pharmacokinetic profile, an appropriate narrow spectrum of activity, reasonable safety, and low cost. Table 2 provides the recommended antimicrobials based on the type of procedure performed^9^. All of these medications are approved by FDA for surgical prophylaxis.

**Table 2.** Recommended antimicrobials for surgical prophylaxis

Type of procedure	Recommended agents	Recommended agents in case of β-lactam allergy
Hernia repair	Cefazolin	Clindamycin, vancomycin
Gastroduodenal (either involving entry into lumen of gastrointestinal tract or not)	Cefazolin	Clindamycin or vancomycin + gentamicin or aztreonam or levofloxacin
Biliary tract Open Laparoscopic high risk, elective^*^ Cefazolin	Cefazolin Cefoxitin Cefotetan Ceftriaxone Ampicillin/sulbactam	Clindamycin or vancomycin + gentamicin or aztreonam or levofloxacin Metronidazole + gentamicin or levofloxacin
Liver transplantation	Piperacillin/tazobactam, cefotaxime + ampicillin	Clindamycin or vancomycin + gentamicin or aztreonam or levofloxacin
Pancreas and pancreas-kidney transplantation	Cefazolin	Clindamycin or vancomycin + gentamicin or aztreonam or levofloxacin
Small intestine Non-obstructed Obstructed	Cefazolin Cefazolin + metronidazole Cefoxitin Cefotetan	Clindamycin + gentamicin or aztreonam or levofloxacin Metronidazole + gentamicin or levofloxacin
Appendectomy for uncomplicated appendicitis	Cefoxitin Cefotetan Cefazolin+ metronidazole	Clindamycin + gentamicin or aztreonam or levofloxacin Metronidazole + gentamicin or levofloxacin
Colorectal^†^	Cefazolin + metronidazole Cefoxitin Cefotetan Ampicillin/sulbactam, Ceftriaxone + metronidazole Ertapenem	Clindamycin + gentamicin or aztreonam or levofloxacin, Metronidazole + gentamicin or levofloxacin

^*^Surgical antimicrobial prophylaxis is recommended for all high-risk patients undergoing elective laparoscopic cholecystectomy. As some high-risk factors, such as conversion to open access and biliary spillage, cannot be predicted before surgery, it is reasonable to administer a single dose of cefazolin to all patients, including those undergoing low-risk elective laparoscopic surgeries

^†^If bowel preparation is an option, it is also advisable to include antibiotic prophylaxis by administering 1 g of neomycin sulfate along with 1 g of erythromycin or 500 mg of metronidazole. However, if bowel preparation is not feasible, there is no need for oral antibiotics, and intravenous administration alone is sufficient.

Patients should be carefully questioned about their history of antibiotic allergies. A documented penicillin allergy also contraindicates the use of β-lactams such as cephalosporins and carbapenems. Table 3 displays the recommended intravenous antibiotic dosages and redosing intervals^11^.

**Table 3.** Recommended intravenous antibiotic dosage and redosing interval

Antibiotic	Intravenous dosage	Redosing interval (h)
Cefazolin	2 g (3 g for patients ≥120 kg)	4
Cefoxitin	2 g	2
Cefotetan	2 g	6
Ceftriaxone	2 g	NA
Cefotaxime	1 g	3
Ampicillin	2 g	2
Ampicillin-sulbactam	3 g	2
Piperacillin/tazobactam	3.375 g	2
Metronidazole	1 g	NA
Ertapenem	1 g	NA
Clindamycin	900 mg	6
Vancomycin	15 mg/kg	NA
Gentamicin	5 mg/kg	NA
Aztreonam	2 g	4
Levofloxacin	500 mg	NA

NA: not applicable.

Maintaining adequate tissue and serum levels of antibiotics throughout the entire duration of the procedure is important. If the surgery exceeds two half-lives of the antimicrobial, it should be readministered^20,45^. Redosing is also necessary if blood loss exceeds 1500 mL. In patients with renal failure, antibiotic excretion is reduced. In such cases, the initial dose administered remains the same, but redosing is not required.

Patients who are receiving antibiotics to treat a distant infection prior to surgery should be administered a different antibiotic if the surgical site pathogen is not susceptible to the current drug being used^10^. If the antibiotic being used to treat the distant infection covers the surgical site pathogen, an additional dose should be given within 60 min before making the surgical incision.

### When should the antibiotic be administered?

At the time of surgical incision, the serum and tissue antibiotic levels should be at least at the minimum inhibitory concentration for the specific drug. This is crucial because most SSIs are caused by gram-positive cocci present on the skin, and the antibiotic must be effective before any contamination occurs.

For most drugs, they should be administered within 60 min prior to the surgical incision^8,45^. However, vancomycin and fluoroquinolones (such as levofloxacin) should be infused within 120 min before the surgical incision, as these drugs require longer infusion times.

Antimicrobial infusions initiated more than 60 min prior to surgery have been associated with a higher rate of SSIs^8,11,25^. Similarly, administering antibiotics too close to the surgical incision has also been associated with increased infection rates. In a real scenario, it could be difficult to initiate antibiotic infusion precisely within this time interval. Some protocols suggest administering it at anesthetic induction.

### How long should the antibiotic be administered for prophylaxis?

There is strong evidence that, for most surgeries, the antibiotic should not be continued after the procedure^8-10,22^. This means that a single dose should be given within 60 min prior to the surgical incision. As previously mentioned, redosing may be necessary in cases of significant blood loss or prolonged surgeries. However, a duration of up to 24 h is also considered acceptable. There is no need to maintain antimicrobial prophylaxis solely due to the presence of drains or central intravenous catheters^11,37^.

In cases of cardiovascular surgery, there is some controversy. Some guidelines recommend a prophylaxis duration of up to 48 h^8^,^9^. It is important to note that these recommendations are based solely on expert opinion, as there are no available data definitively defining the optimal duration. However, it appears that there is no advantage in extending prophylaxis beyond 24 h.

### Antibiotic prophylaxis in obese patients

Antibiotic prophylaxis requires an effective spectrum, appropriate pharmacokinetics, low toxicity, and an appropriate duration, as well as maximum concentration in the tissues during the incision^16,17,21^. However, these recommendations were based on healthy and non-obese patients.

The use of antibiotics and their distribution in obese patients, particularly in the context of obese surgical literature, lacks sufficient evidence. Less is known about the pharmacokinetics of antibiotics in patients with a body mass index (BMI) above 40 kg/m^2 23^.

Obese patients absorb, distribute, metabolize, and excrete drugs differently. The relationship between body size, physiological variables, and pharmacokinetic parameters has been evaluated in the obese population^15,23^. Some physiological changes characteristic of morbid obesity has implications for drug kinetics, including increased cardiac output, total blood volume, renal clearance, fatty deposition in the liver, and alterations in plasma proteins.

The incidence of SSI in obese patients is high, and the current recommendations for antibiotic prophylaxis are inadequate^2^. In this patient population, SSIs tend to have significant morbidity.

### Which is the best route of administration?

The best route of administration is intravenous. At the time of incision, the tissue and serum antibiotic levels should be at least at the minimum inhibitory concentration for the drug. Intravenous administration is fast, predictable, and reliable. The most common method is bolus infusion, although recent evidence suggests that continuous infusion may be superior^21,44^.

### Bolus versus continuous infusion

Recent studies have shown better results for the use of continuous prophylactic antibiotic infusion when compared to intermittent bolus infusion^36^. Naik etal., in a randomized study, evaluated intermittent bolus infusion of cefazolin (2 g every 4 h) compared with continuous infusion (500 mg/h)^36^. They demonstrated that continuous intraoperative infusions of cefazolin provide better plasma concentrations, even with lower infusion doses.

Skhirtladze-Dworschak etal. compared antibiotic prophylaxis with cefuroxime using intermittent bolus and continuous infusion methods, assessing their serum and subcutaneous tissue concentrations^44^. They observed higher concentrations of cefuroxime in both plasma and subcutaneous tissue when cefuroxime was administered continuously and concluded that patients who received the antibiotic through continuous infusion had higher concentration measurements.

Ferraz etal. conducted a study comparing the continuous infusion of cefazolin with ampicillin/sulbactam and ertapenem in bariatric patients, evaluating their effects on the incidence of SSI^21^. The study analyzed the infection rate and its association with age, gender, preoperative weight, BMI, and comorbidities. The results showed that the rates of SSI were 4.16% in the group prophylactically treated with ampicillin/sulbactam, 1.98% for ertapenem, and 1.55% for continuous cefazolin. The authors concluded that the prophylactic use of cefazolin in continuous infusion yields very promising results.

Shoulders etal. studied the impact of intermittent in bolus cefazolin prophylaxis versus continuous infusion on the incidence of SSIs^43^. A total of 516 adult patients received cefazolin intraoperatively, with 284 receiving intermittent bolus infusion and 232 receiving continuous infusion. The study found that superficial SSIs were significantly reduced in patients who received antibiotic prophylaxis in the form of continuous infusion (2.8% in intermittent bolus versus 0.4% in continuous, p=0.039).

There are very limited data on topical solutions, except in the field of ophthalmology. Some older studies have shown efficacy compared to placebo^35,39^. A meta-analysis encompassing 13 RCTs compared the efficacy and safety of topical antibiotics with non-antibiotic agents in preventing SSI but demonstrated no reduction in the occurrence of SSI^13^. When compared with intravenous administration, topical solutions have inferior results in terms of SSI rates. However, further trials are needed to assess the effectiveness of topical solutions in high-risk surgeries or selected patient groups.

In colorectal procedures, both oral and intravenous antimicrobials can be used. The necessity of intravenous antibiotics is undisputed, but the question is whether they should be used alone or in combination with oral drugs. Oral antimicrobials are commonly administered alongside bowel preparation, which is a highly debated topic in the literature. Major perioperative care protocols such as Enhanced Recovery After Surgery and Acerto recommend limiting bowel preparation^6,19,24,26^. The rationale behind this is to reduce hydroelectrolytic imbalance and the need for intravenous fluids during the perioperative period^24,26^. The concept of no bowel preparation in elective colorectal surgery is supported by a randomized trial from Finland (MOBILE trial), which showed no difference in terms of SSIs and overall morbidity between mechanical bowel preparation, oral antibiotic bowel preparation, and no bowel preparation^32^.

The most studied regimens include three doses of neomycin sulfate plus three doses of erythromycin or metronidazole. If bowel preparation is an option, it is recommended to include both oral and intravenous antibiotic prophylaxis^14,24,38^. However, if colon preparation is not performed, there is no need for oral antibiotics, and only intravenous administration is necessary.

### What are the main problems related to antibiotic prophylaxis in surgery?

Most of the problems related to antibiotic prophylaxis in surgery are caused by inadequate administration, leading to higher rates of SSIs, the development of resistant bacteria, and *C. difficile* infection.

One study showed an appropriateness rate of almost 70% for prophylactic antibiotic use^42^. In 41% of these cases, the antibiotic was necessary but not used, or it was unnecessary; in 29% of cases, the prescription took longer than necessary. The same study demonstrated that implementing an antibiotic stewardship program reduced the appropriateness rate to 36%. Major perioperative care protocols emphasize the importance of implementing an adequate antibiotic prophylaxis protocol^19,30,33^.

SSIs are associated with a 2–11-fold increase in mortality and prolonged hospital stays^4^. They account for up to 20% of hospital-acquired infections^27^. Most SSIs are caused by resistant bacteria.

Multidrug-resistant bacteria are responsible for more than 2.8 million infections in the United States every year^46^. They cause at least 35,000 deaths and result in $20 billion in healthcare expenditures. Data from the National Nosocomial Infections Surveillance (NNIS) System identified *S. aureus* as the most common cause of SSIs in the United States, accounting for 22.5% of infections. Of these, 49% were caused by methicillin-resistant *S. aureus* (MRSA)^29^. Other common resistant bacteria include extended-spectrum β-lactamase (ESBL) *Escherichia coli*, extended-spectrum β-lactamase and carbapenemase-producing (KPC) *Klebsiella pneumoniae*, *Proteus mirabilis* (ESBL), and *Pseudomonas aeruginosa*.

### What is the recommended prophylaxis in the case of resistant bacterial colonization?

Currently, routine screening and eradication for patients colonized by resistant bacteria are not recommended^21^. However, if a patient is colonized, there are several strategies that can be adopted, with most of them described for patients colonized by MRSA.

The most common strategy is the use of topical 2% mupirocin administered three times a day intranasally, along with daily baths using 2% chlorhexidine for 5 days^8,9^. Another strategy is the use of vancomycin^41^. However, it is important to consider that vancomycin is not effective against methicillin-susceptible *S. aureus*. In such cases, some authors recommend combining vancomycin with another drug that targets the expected surgical-site pathogens. For example, cefazolin can be added for gram-positive cocci^12^.

## CONCLUSION

The correct use of antibiotic prophylaxis in gastrointestinal surgery is essential for achieving the best surgical results, minimizing the incidence of SSIs, reducing morbidity and mortality rates, and lowering costs. Therefore, surgeons should be familiar with administering the appropriate antibiotic to the right patient, at the optimal time, in the correct dosage, and for the appropriate duration. Adherence to institutional protocols should be encouraged as it can significantly improve surgical outcomes.
